# Privacy-Preserving Hybrid GA–LSTM Ensemble for Typhoid Detection Using Optimised Clinical Feature Selection

**DOI:** 10.3390/biomedicines14051010

**Published:** 2026-04-29

**Authors:** Karim Gasmi, Afrah Alanazi, Sahar Almenwer, Sarah Almaghrabi, Hamoud Alshammari, Kais Khaldi, Hassen Chouaib

**Affiliations:** 1 Department of Computer Science, College of Computer and Information Sciences, Jouf University, Sakaka 72388, Saudi Arabia; smalmenwer@ju.edu.sa (S.A.); kkhaldi@ju.edu.sa (K.K.); 2 Department of Information System, College of Computer and Information Sciences, Jouf University, Sakaka 72388, Saudi Arabia; aoalenzy@ju.edu.sa (A.A.); hhalshammari@ju.edu.sa (H.A.); 3 Department of Information Technology, College of Computing and Information Technology at Khulais, University of Jeddah, Jeddah 23218, Saudi Arabia; ssalmaghrabi@uj.edu.sa; 4 Department of Physics, College of Science, Jouf University, Sakaka 72341, Saudi Arabia; hchouaib@ju.edu.sa

**Keywords:** SDG 3, deep learning, optimisation, feature selection, genetic algorithm, typhoid detection

## Abstract

**Background/Objectives:** Typhoid fever remains a major public health challenge in many low-income countries, where overlapping clinical symptoms and the limited reliability of conventional diagnostic procedures hinder accurate diagnosis. This study aims to develop a reliable and efficient diagnostic framework that automates typhoid fever detection from clinical data while preserving patient privacy. **Methods:** To achieve this objective, we propose a hybrid framework combining genetic algorithm (GA)–based feature selection, a Convolutional Neural Network–Long Short-Term Memory (CNN–LSTM) deep learning classifier, and federated learning. The GA identifies the most informative clinical features, reducing redundancy and computational complexity. The selected features are then used to train a CNN–LSTM model in a federated learning setup using the Federated Averaging (FedAvg) algorithm, enabling collaborative model training across multiple clients without sharing raw patient data. **Results:** Experimental results show that the proposed framework achieves 92% accuracy, with a strong F1-score and satisfactory sensitivity. Compared to models trained on the full feature set, the proposed approach requires less memory and shorter training time, while maintaining balanced performance under class imbalance. **Conclusions:** These results demonstrate that integrating evolutionary feature selection, deep sequential learning, and federated training provides an effective and privacy-aware solution for multi-class typhoid fever diagnosis. The proposed framework is particularly suitable for clinical environments with limited data access and constrained resources.

## 1. Introduction

Typhoid fever remains one of the most severe epidemics affecting public health in low- and middle-income countries. Annually, it results in 11 to 20 million infections, along with significant morbidity and mortality. The disease is induced by the bacterium Salmonella enterica serovar Typhi. Due to its frequent transmission through contaminated food and beverages, it is imperative to identify it promptly and accurately to prevent further spread. Typhoid fever is of significant importance in medicine, despite frequent misdiagnosis or complete omission. The principal reason for this is that its symptoms resemble those of other febrile diseases, such as dengue and malaria. Due to the similarity of clinical manifestations among several disorders, such as fever, abdominal pain, headache, fatigue, and emesis, clinical diagnosis lacks reliability without confirmatory testing. Due to the lack of adequate laboratories in many endemic regions, medical workers occasionally utilise diagnostic methods with inherent limitations.

Despite the utilisation of conventional diagnostic techniques such as the Widal agglutination test for more than a century, numerous studies have demonstrated their insufficient sensitivity and specificity, frequently resulting in erroneous treatment decisions [1]. Recent research has underscored the imperative need for improved diagnostic tools that integrate clinical symptoms, laboratory findings, and data-driven statistical patterns to help medical personnel distinguish typhoid fever from other febrile infections [2]. Consequently, machine learning (ML) and artificial intelligence (AI) have garnered considerable interest as prospective instruments to enhance diagnostic precision and facilitate earlier detection in healthcare settings with limited resources [3,4].

The preliminary research on the application of machine learning for diagnosing typhoid fever demonstrated that even rudimentary classifiers can yield substantial diagnostic criteria from clinical characteristics. Oguntimilehin et al. utilised rule-based classifiers that produced interpretable decision rules from clinical parameters, pioneering one of the earliest machine learning approaches for diagnosing typhoid illness [5].

In later research, supervised learning techniques, including support vector machines (SVMs), were utilised to differentiate among amoebic dysentery, typhoid fever, and malaria. Upon training on pertinent clinical characteristics, it was demonstrated that machine learning models could precisely categorise patients with febrile illness [6]. An investigation was conducted into artificial neural network methodologies, including multilayer perceptrons, for mobile diagnostic systems. However, these models frequently relied on manual feature engineering and exhibited insufficient robustness across diverse populations [7]. The preliminary efforts demonstrated the feasibility of machine learning for typhoid diagnosis, while also highlighting issues of feature redundancy, overfitting, and the generalisability of diagnostic models.

In recent years, significant advances have been made in developing sophisticated models, particularly in ensemble learning and metamodel architectures [8,9]. While this research collectively illustrates the potential of ensemble models to improve diagnostic accuracy, most rely on extensive, unrefined feature sets and do not employ evolutionary feature selection or systematic classifier fusion.

Deep learning (DL) has emerged as a robust method for modelling complex, nonlinear interactions in clinical and epidemiological data. This progress has occurred concurrently with the gains made in traditional machine learning (ML). Studies on time-series modelling [10,11] indicate that neural network methodologies outperform conventional statistical models, such as ARIMA and exponential smoothing, in forecasting trends in typhoid incidence. Nevertheless, while these findings demonstrate the representational effectiveness of deep models, they have not yet been directly employed for patient-level diagnosis utilising structured clinical data. The research reveals a lack of applications of LSTM architectures in typhoid classification, despite the widespread utilisation of deep learning in biological contexts and chronic disease prediction [12].

Current studies also lack a feature selection component. Clinical datasets likely contain elements that are superfluous, noisy, or of little utility. These characteristics may deteriorate performance, increase computational expenses, and complicate comprehension. No studies specifically addressing typhoid have utilised automated feature selection techniques, such as a genetic algorithm (GA), despite several articles emphasising the importance of feature engineering and model optimisation in clinical machine learning. Similarly, none of them utilise GA-driven modifications to the ensemble weights to combine deep and conventional classifiers.

The novelty of this work lies in the joint integration of three complementary components within a unified diagnostic framework: (i) a Genetic-Algorithm-based wrapper feature selection strategy that reduces the original clinical feature space to a compact subset of only six variables, (ii) a CNN–LSTM hybrid model capable of learning both local feature interactions and temporal dependencies, and (iii) a federated learning paradigm that enables decentralised collaborative model training across distributed healthcare environments. In contrast to existing typhoid diagnostic systems based on centralised ensemble models such as XGBoost, Random Forests, or stacked metamodels operating on full feature sets, the proposed approach emphasizes compactness, deep sequential representation learning, and decentralized deployment.

The remainder of this paper is structured as follows: Section 2 reviews recent studies and approaches related to typhoid detection. Section 2.4 presents our proposed two-stage framework, including centralised genetic-algorithm-based feature selection, preprocessing steps, and the federated CNN–LSTM architecture. Section 3 describes the experimental setup, detailing the dataset, evaluation metrics, and baseline configurations. Section 4 reports the results, comparing individual models with the federated approach and assessing the impact of optimised feature subsets. Finally, Section 4 summarises the key contributions and outlines potential directions for future research.

## 2. Materials and Methods

Various computational strategies for detecting typhoid illness include early expert systems, conventional machine learning models, ensemble metamodels, and time-series forecasting of typhoid incidence. The primary objective of this study is to address the limited sensitivity and specificity of routine serological tests, as well as the absence of laboratory facilities in many low- and middle-income countries. This section emphasises the key findings from published papers. Our emphasis is on (i) early expert systems and fuzzy-logic decision support, and (ii) learning models predicated on symptoms and coinfections.

### 2.1. Early Expert Systems and Fuzzy-Logic Decision Support

Oguntimilehin et al. [5] presented an instance of a pioneering decision-support system for typhoid fever that utilised machine learning. Oguntimilehin developed a clinical diagnostic framework capable of deriving decision-making principles from medical data and symptom descriptions. Their approach aimed to assist physicians operating in resource-limited settings by autonomously proposing potential diagnoses based on observed and unobserved symptoms. The research suggested that data-driven rules may potentially mimic clinical reasoning; nevertheless, it utilised only rudimentary prediction models and failed to integrate advanced feature selection or deep learning methodologies.

Samuel and coauthors [13] developed a web-based decision support system employing fuzzy logic to assist physicians in diagnosing typhoid fever. That system was founded on the requirement for more intelligent and adaptive systems. Their methodology employed fuzzy rules and membership functions to transform information provided by specialists into a format that the algorithm could utilise to interpret ambiguous verbal symptoms. The fuzzy-logic engine achieved a diagnostic accuracy of approximately 94%, as reported by the authors, owing to its superior handling of uncertainty in clinical data compared with rigid thresholding methods. The system, conversely, did not utilise statistical learning on large datasets, thereby limiting its ability to identify novel patterns or to autonomously adapt to shifts in clinical practice.

In diagnosing typhoid fever, these preliminary data suggested that computational decision support could improve clinical judgement. However, they were constrained since they excluded ensemble or deep learning models, systematic data-driven learning, and comprehensive feature sets that did not incorporate feature selection.

### 2.2. Symptoms-Based Model Learning for Malaria–Typhoid Coinfection

Numerous studies have demonstrated that typhoid fever and malaria frequently co-occur and share similar clinical manifestations. This complicates the differentiation between them in areas where they are prevalent. Aminu et al. in [6] introduced a predictive symptom-based methodology utilising support vector machines (SVMs) to enhance classification precision in cases of malaria and typhoid coinfection. They developed a model of the same symptomatology for malaria and typhoid. It successfully cross-validated on both diseases with high accuracy. The authors noted that the sample size was insufficient, the characteristics lacked diversity, and the data were from a single institution, rendering the results difficult to generalise to other contexts.

Odion et al. [9] enhanced web-based diagnostics for febrile illnesses by demonstrating the capability of a machine learning system to detect typhoid and malaria. Utilising XGBoost, they developed a Typhoid Binary Classifier (TBC) that achieved approximately 96% accuracy in classifying typhoid. Although XGBoost showed efficacy, the study examined solely one ensemble technique.

These findings demonstrate that, when trained on meticulously curated clinical datasets, symptom-based support vector machines (SVMs), probabilistic algorithms, and tree-based ensembles can distinguish among malaria, typhoid, and co-infection. They often utilise predefined feature sets and processes that incorporate a singular model. This indicates that both feature selection and multi-classifier fusion may be superior.

Nishat et al. [14] proposed a modern and comprehensive approach for diagnosing typhoid fever utilising clinical parameters. The authors of that work developed a lightweight metamodel. This metamodel was constructed by integrating various core learners, including Decision Tree, Gradient Boosting, K-Nearest Neighbours, Linear Discriminant Analysis, Logistic Regression, Multi-Layer Perceptron (MLP), Naïve Bayes, support vector machine, and XGBoost. Nishat et al. utilised a Stacking Classifier as the meta-learner, with LightGBM serving as the final estimator. A meticulously selected clinical dataset was utilised to train the neural network. This dataset included demographic information, symptoms, and laboratory test results, including haemoglobin, platelet count, and electrolyte values. The results indicated that the metamodel outperformed all individual classifiers. It exhibited superior accuracy and F1-score, performing effectively in both classes (typhoid positive and typhoid negative) [14]. Moreover, comprehensive confusion matrices and performance metrics indicated that the metamodel exhibited less errors in identifying both categories compared to the individual models. This illustrates the efficacy of meta-ensembles in clinical classification challenges.

However, even in this avant-garde study, certain considerations remain necessary. Initially, each model was trained on the complete array of available clinical characteristics. The authors stated that redundant or low-quality characteristics could impair performance and hinder comprehension. Determining the selection of characteristics in the pipeline was challenging. The metamodel comprised only standard machine learning classifiers. It did not employ deep learning models such as LSTM or BiLSTM, which are capable of learning more intricate relationships among clinical scenarios. Third, the stacking technique did not optimise the ensemble of features in the metamodel.

A recent study domain enhanced ensemble learning by utilising multiple forms of clinical data. Support vector machines, Gaussian naive Bayes, and decision trees were employed in conjunction with LightGBM, according to their respective methodologies. They achieved a precision of 0.99 using k-fold validation on a bespoke dataset. This indicates that the technique could be beneficial in resource-constrained clinics; nevertheless, additional testing is required prior to wider adoption [14]. The Coalition Against Typhoid reaffirmed the conclusions in its summary for practitioners and emphasised that these findings could be applicable in a broader array of contexts, provided workflow integration and multi-site evaluation were addressed [14]. Semuyiga et al. [15] proposed a multi-task XGBoost model that utilised routinely collected clinical profiles to predict typhoid, treatment outcomes, duration, and a resistance proxy score. This system was capable of more than mere binary detection. The SHAP explanations identified critical characteristics and relevant patient groups, while the counterfactual medication simulation demonstrated the viability of model-informed antibiotic ranking. It is crucial to recognise that peer review and external validation are vital.

This collection of work on machine learning predominantly addresses two principal concerns. Initially, clinically accessible components such as symptom checklists, temperature ranges, complete blood count indices, and electrolyte levels may provide significant differentiation before culture data become available. The second most critical unanswered question is generalisation. The majority of reports employ internal cross-validation, lacking sufficient external, multi-site evaluation, calibration to local prevalence, or clearly defined operating points (precision/recall trade-offs) aligned with clinical utility and antibiotic stewardship [14,16,17].

Given these shortcomings, it is essential to incorporate metaheuristic feature selection and deep learning into typhoid diagnosis frameworks, as analysed in the present study.

### 2.3. Summary and Research Gap

Several significant tendencies are evident in the works examined. Early expert systems and fuzzy-logic frameworks demonstrated that computational tools could replicate clinical reasoning. Nonetheless, these frameworks did not employ comprehensive datasets or modern learning techniques [5,13].

Symptom-based machine learning systems for malaria–typhoid coinfection have shown that support vector machines (SVMs) and probabilistic models can effectively classify febrile illnesses, provided sufficient symptom data are available. It is customary for them to process all input features equally and employ a single classifier [9]. Recent research on metamodels for typhoid detection using clinical factors indicates that aggregating multiple machine learning models yields superior performance. Nevertheless, this is accomplished by relying on extensive feature sets without explicit feature selection or sophisticated sequence modelling [14].

Extensive research on machine learning in clinical decision support underscores the importance of accurate feature selection, interpretable models, and adaptability to resource-limited settings [12,18].

Nonetheless, none of the studies cited in the relevant literature combine feature selection using genetic algorithms (GAs) with deep learning methods, particularly long short-term memory (LSTM) and federated learning, for the identification of typhoid illness.

Consequently, no hybrid framework exists that (i) independently employs genetic algorithms (GAs) to identify the most pertinent clinical parameters, (ii) utilises a long short-term memory (LSTM) classifier to demonstrate nonlinear interactions among the selected features, and (iii) implements federated learning to maintain data privacy. The aim of the approaches outlined in this work is to improve existing research on AI-assisted typhoid diagnosis and to address the identified deficiencies.

Unlike existing typhoid diagnostic approaches that rely on full feature sets and centralised ensemble models such as XGBoost or stacking classifiers, the proposed framework integrates evolutionary feature selection with deep sequential modelling and federated learning. This combination enables dimensionality reduction, improved representation learning, and privacy-aware deployment, which are not jointly addressed in previous studies.

### 2.4. Proposed Approach for Typhoid Detection

The suggested diagnostic architecture comprises a feature selection approach utilising a genetic algorithm (GA), a CNN–LSTM deep classifier, and an enhanced ensemble mechanism. This section provides a mathematical characterisation of each component and an overarching understanding of their interrelations.

**Figure 1:**
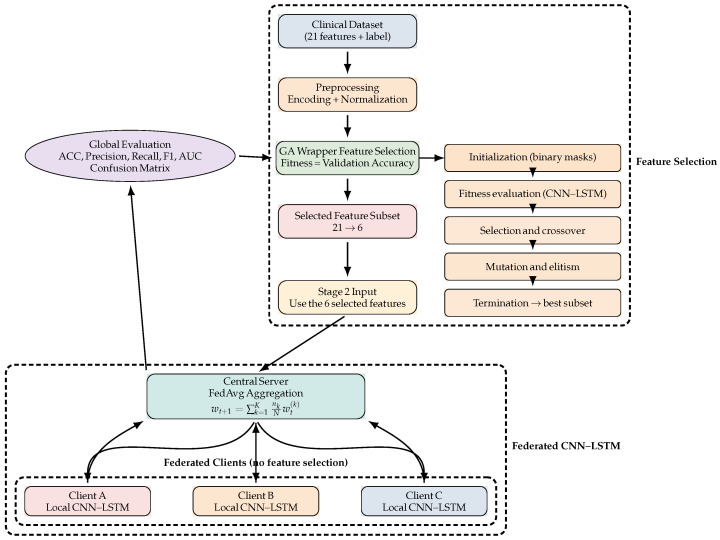
Two-stage typhoid detection framework. **Stage 1** performs a one-time centralised GA-based wrapper feature selection to reduce the clinical feature space from 21 to 6 parameters. The selected features are then fixed and used as input to **Stage 2**, where a CNN–LSTM model is trained in a federated learning setting using FedAvg without sharing raw patient data, followed by global evaluation on a held-out test set.

The methodological design seeks to rectify three common shortcomings in typhoid prediction models: (i) the incorporation of clinical features that are either extraneous or unreliable; (ii) the inadequacy of simplistic models to effectively capture nonlinear clinical patterns; and (iii) the absence of patient data confidentiality. The suggested approach employs genetic algorithms (GAs) for feature reduction and ensemble optimisation, alongside long short-term memory (LSTM) for advanced representation learning. This ensures that the results are both comprehensible and precise and can be applied to other clinical datasets. All steps are presented in the Figure 1.

#### 2.4.1. Proposed Algorithm

In this section, we summarise the overall workflow of the proposed GA–LSTM-based typhoid diagnosis framework in the form of pseudocode. The Algorithm 1 takes as input a labelled clinical dataset in CSV format and outputs a trained model that predicts whether a patient has typhoid.
**Algorithm 1** Two-stage GA feature selection and federated CNN–LSTM typhoid detection framework**Require:** Clinical dataset $X\in R^{n\times d}$, labels $y\in {\left\{0,1\right\}}^{n}$, number of clients *K*, GA parameters $\left(P,G_{\max},\mu \right)$, federated rounds *R*, local epochs *E*, batch size *B* **Ensure:** Selected feature mask $m^{*}$, trained global CNN–LSTM model parameters $w_{R}$   1:**Preprocessing:**  2:   Clean missing or inconsistent records in *X*  3:   Encode categorical attributes (e.g., label/one-hot encoding)  4:   Normalise/standardise numerical features  5:**Stage 1: One-time centralised GA-based feature selection**  6:   Randomly initialize a population of binary masks ${\left\{m^{\left(j\right)}\right\}}_{j=1}^{P}$, $m^{\left(j\right)}\in {\left\{0,1\right\}}^{d}$  7:**for** g=1 to $G_{\max}$ **do**  8:    **for** each chromosome $m^{\left(j\right)}$ in the population **do**  9:        Construct subset $X_{m^{\left(j\right)}}=X⊙m^{\left(j\right)}$10:       Split $X_{m^{\left(j\right)}}$ into training/validation sets11:      Train a CNN–LSTM classifier on the training set for *E* epochs with batch size *B*12:       Evaluate validation accuracy ${\mathrm{Acc}}_{val}\left(m^{\left(j\right)}\right)$13:       Set fitness $\mathrm{Fitness}\left(m^{\left(j\right)}\right)={\mathrm{Acc}}_{val}\left(m^{\left(j\right)}\right)$14:   Select parents (tournament/roulette-wheel) based on fitness15:   Apply crossover to generate offspring masks16:   Apply mutation to offspring masks with probability μ17:   Form the next population (optionally keep elite masks)18:Select the best mask $m^{*}=\arg\max_{m^{\left(j\right)}}\mathrm{Fitness}\left(m^{\left(j\right)}\right)$19:Reduce the dataset: $X^{*}=X⊙m^{*}$20:**Stage 2: Federated CNN–LSTM training using fixed selected features**21:Partition $X^{*}$ and *y* across *K* clients: ${\left\{\left(X_{k}^{*},y_{k}\right)\right\}}_{k=1}^{K}$ *(IID or non-IID)*22:Initialize global model parameters $w_{0}$ for CNN–LSTM23:**for** t=0 to R−1 **do**24:    Server broadcasts current global parameters $w_{t}$ to all clients25:    **for** each client k=1 to *K* **in parallel do**26:        Initialize local parameters $w_{t}^{\left(k\right)}\leftarrow w_{t}$27:        Train CNN–LSTM locally on $\left(X_{k}^{*},y_{k}\right)$ for *E* epochs with batch size *B*28:        Send updated parameters $w_{t+1}^{\left(k\right)}$ (or model update $\Delta w_{t}^{\left(k\right)}$) to the server29:    Server aggregates updates with FedAvg:30:       $w_{t+1}\leftarrow \sum_{k=1}^{K}\frac{n_{k}}{N}\;w_{t+1}^{\left(k\right)}$31:Evaluate the final global model $w_{R}$ on a held-out test set (ACC, Precision, Recall, F1, AUC)32:**return** $m^{*}$ and $w_{R}$

#### 2.4.2. Dataset Representation

The dataset was obtained from a publicly available repository (Kaggle), containing anonymised clinical records. As no personally identifiable information was included, formal ethical approval and informed consent were not required for this study.

A single large clinical dataset was used in this study. The Typhoid Multi-Class dataset comprises 31,087 anonymised patient records, as presented in the Table 1 categorised into four diagnostic classes: Normal or No Typhoid, Acute Typhoid Fever, Relapsing Typhoid, and Complicated Typhoid. The class distribution is highly imbalanced, with Normal or No Typhoid cases constituting approximately three quarters of the dataset, while Complicated Typhoid representing fewer than 5% of samples. This skew reflects real-world clinical prevalence and was preserved during model training.

The dataset is a large and diverse dataset comprising twenty-one variables. It includes clinical symptoms, environmental exposures, behavioural risk factors, and the patient’s medical history. This dataset has a lot of information about different things, such as how long the fever lasts, headaches, stomach pain, vomiting, general weakness, sanitation conditions, the quality of the water source, eating street food, seasonal weather patterns, and signs of infections that are still happening in the community, like dengue or COVID-19 outbreaks. The dataset also has information on the person’s medical history. This includes information on any prior cases of typhoid fever and any antibiotics the person has received. There are four ways to diagnose typhoid: standard or no typhoid, relapsing typhoid, acute typhoid fever, and complex typhoid. This multi-class structure enables assessment of the proposed framework on a more complex classification problem. In this case, it is important to distinguish between how severe the condition is and how quickly it worsens, rather than simply determining whether it is present. The dataset includes both male and female patients across a wide adult age range. Gender distribution is approximately balanced.

Missing values were handled during preprocessing. Numerical features were scaled using statistics computed from the training data, while categorical attributes were encoded using robust encoders that safely handled missing or unseen values.

To emulate multiple hospitals, the dataset was partitioned into federated clients under both IID (Independent and Identically Distributed) and non-IID settings.

Although all clients originated from the same dataset, non-IID splits introduced heterogeneous class distributions to approximate inter-hospital variability.

#### 2.4.3. Feature Extraction Strategy

This investigation utilised a clinical dataset comprising several tabular variables. These factors encompassed categorical attributes, ordinal symptom indicators, serological test outcomes, environmental descriptors, and quantitative clinical measurements. We utilised a standard tabular feature-extraction technique that directly used the clinical parameters used at the start to keep the model clear and ensure it matched real-world clinical data. We did not use more complicated ways to learn how to represent things.

The preprocessing method included handling missing data (removing or imputing as needed), encoding categorical variables using methods such as one-hot or label encoding, and scaling numerical features using either standardisation or min–max normalisation. During these stages, the GA and neural network components were tested to ensure they performed well on input characteristics in good condition. We carefully examined and addressed any severe outliers we observed to ensure the learning process was not interrupted.

##### Encoding of Categorical and Ordinal Variables

Categorical features in the dataset, such as symptom presence, sanitation conditions, patient history indicators, and laboratory test categories, were transformed into numerical representations using **one-hot encoding**. Ordinal symptom indicators were preserved in their natural ordered form to retain severity-related information.

Let $x_{j}$ denote a categorical or ordinal feature. After encoding, each feature was represented as a numerical vector:

$${\tilde{x}}_{j}\in R^{q_{j}},$$

where $q_{j}>1$ corresponds to the one-hot encoded dimensionality of feature $x_{j}$. All encoded features were concatenated to form a tabular feature vector:

$$\tilde{x}=[{\tilde{x}}_{1}∥{\tilde{x}}_{2}∥\cdots ∥{\tilde{x}}_{d}].$$

This encoding strategy ensured simplicity, interpretability, and compatibility with both classical machine learning models and deep learning architectures.

##### Normalisation of Numerical Features

Continuous numerical features, including laboratory measurements and physiological indicators, were standardised using standard scaling to mitigate scale disparities and accelerate training convergence. Given a numerical feature $x_{j}$, normalization was performed as:

$$x_{j}^{\mathrm{norm}}=\frac{x_{j}-\mu_{j}}{\sigma_{j}},$$

where $\mu_{j}$ and $\sigma_{j}$ denote the mean and standard deviation estimated from the training data; the same transformation was applied consistently across validation and test sets.

#### 2.4.4. GA-Based Feature Selection

Feature selection is a critical step in clinical prediction tasks, as medical datasets often contain redundant, weakly informative, or noisy parameters that can degrade model performance and increase computational cost. Genetic algorithms (GAs) provide an effective evolutionary search strategy for exploring large combinatorial feature spaces and identifying clinically relevant variables. In this work, GA was employed in a single, centralised phase to identify the most informative subset of clinical parameters prior to model training. This strategy reduced overfitting, improved efficiency, and enhanced interpretability by highlighting the features that most contributed to typhoid fever detection.

GA explores the space of binary feature masks defined as:

$$m=\left[m_{1},m_{2},\ldots ,m_{d}\right],\;m_{i}\in \left\{0,1\right\},$$

where each gene $m_{i}$ indicates whether the corresponding clinical parameter is selected. Applying a mask *m* to the feature matrix yields:

$$X_{m}=X⊙m.$$

##### Feature Representation for GA Selection and CNN–LSTM Classification

After encoding and normalisation, all features were combined into a unified tabular representation that served as input to the GA-based feature selection stage. Each original clinical parameter corresponded to a gene in the GA chromosome, enabling the algorithm to select or discard features at the parameter level directly.

The reduced feature subset identified by GA was then reshaped as required and provided as input to the CNN–LSTM classifier. In this setting, convolutional layers learned local interactions among selected clinical features, while the LSTM component modelled higher-order dependencies across the feature sequence. This design allowed the framework to leverage deep learning capabilities while preserving the interpretability and efficiency of classical tabular feature processing.

#### 2.4.5. Fitness Function

Each candidate feature subset was evaluated using a wrapper-based fitness function that directly reflected classification performance. For each chromosome, a CNN–LSTM classifier was trained on the selected feature subset and evaluated on a validation set. The fitness function was defined as:

$$\mathrm{Fitness}\left(m\right)={\mathrm{Acc}}_{\mathrm{val}}\left(m\right),$$

where ${\mathrm{Acc}}_{\mathrm{val}}\left(m\right)$ denotes the validation accuracy obtained using features $X_{m}$. This formulation prioritises predictive performance while allowing the GA to naturally converge to compact and informative feature subsets.

##### Genetic Operators

The GA population was evolved using standard evolutionary operators. Selection favours individuals with higher fitness, crossover recombines parental feature masks to explore new subsets, and mutation introduces stochastic perturbations to avoid premature convergence. These operators collectively enable efficient exploration of the high-dimensional clinical feature space.

The GA iterated until convergence or a maximum number of generations was reached, and the best-performing feature mask $m^{*}$ was retained. The reduced dataset $X_{m^{*}}$ was then fixed and used throughout all subsequent experiments.

#### 2.4.6. CNN–LSTM Classifier

After GA-based feature selection, the reduced clinical feature vector was used to train a CNN–LSTM classifier. Let

$$x=\left[x_{1},x_{2},\ldots ,x_{d^{\prime }}\right]\in R^{d^{\prime }}$$

denote the selected clinical features for a patient, where $d^{\prime }$ is the number of retained parameters. The feature vector is reshaped as a one-dimensional sequence and passed through convolutional and recurrent layers to capture local interactions and higher-order dependencies among clinical variables.

##### Convolutional Feature Extraction

The convolutional layer applies a set of one-dimensional filters over the input feature sequence to learn local patterns among neighbouring clinical parameters. For a convolutional filter *k* of size *r*, the convolution operation at position *t* is defined as:

$$c_{t}^{\left(k\right)}=\sigma \;\left(\sum_{i=0}^{r-1}w_{i}^{\left(k\right)}\;x_{t+i}+b^{\left(k\right)}\right),$$

where $w^{\left(k\right)}$ and $b^{\left(k\right)}$ denote the filter weights and bias, respectively, and σ(·) is a nonlinear activation function. Applying multiple filters produces a set of feature maps capturing diverse local interactions:

$$C=[c^{\left(1\right)},c^{\left(2\right)},\ldots ,c^{\left(K\right)}].$$

#### 2.4.7. Classification Using LSTM and CNN–LSTM

**Figure 2:**
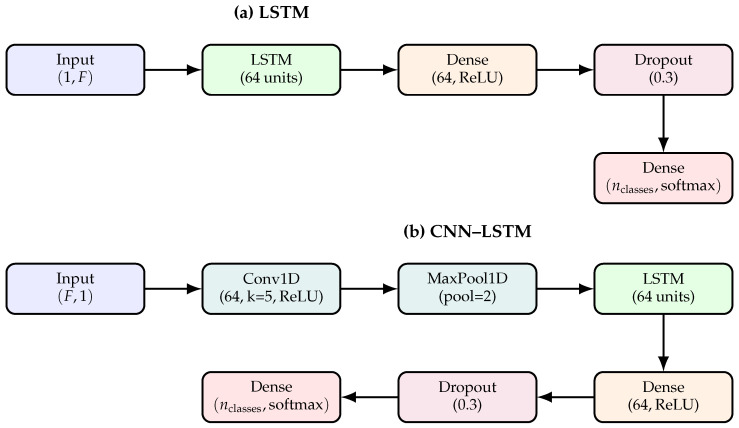
Architectures for sequence classification. (**a**) LSTM model with a serpentine layout for compact presentation. (**b**) CNN–LSTM hybrid model combining convolutional feature extraction with temporal modelling.

For sequence classification, we employed two architectures: a standard **LSTM** and a hybrid **CNN–LSTM** model, this two architectures presented by the Figure 2. The LSTM network is designed to capture temporal dependencies in sequential data. At the same time, the CNN–LSTM model combines convolutional layers for local feature extraction with LSTM layers for modelling long-term dependencies.

##### LSTM Model

The LSTM model processes input sequences of shape (1,F), where *F* is the number of features. The architecture consists of:

- An LSTM layer with 64 units to learn temporal patterns.
- A fully connected layer with ReLU activation for non-linear transformation.
- A dropout layer (rate =0.3) to reduce overfitting.
- A final dense layer with softmax activation for multi-class classification.

The LSTM cell updates its states using the following equations:

$$f_{t}=\sigma \left(W_{f}x_{t}+U_{f}h_{t-1}+b_{f}\right),\;i_{t}=\sigma \left(W_{i}x_{t}+U_{i}h_{t-1}+b_{i}\right),$$

$${\tilde{c}}_{t}=\tanh\left(W_{c}x_{t}+U_{c}h_{t-1}+b_{c}\right),\;c_{t}=f_{t}⊙c_{t-1}+i_{t}⊙{\tilde{c}}_{t},$$

$$o_{t}=\sigma \left(W_{o}x_{t}+U_{o}h_{t-1}+b_{o}\right),\;h_{t}=o_{t}⊙\tanh\left(c_{t}\right),$$

where $x_{t}$ is the input at time *t*, $h_{t}$ is the hidden state, $c_{t}$ is the cell state, σ is the sigmoid activation, and ⊙ denotes element-wise multiplication. Here, $o_{t}$ denotes the output gate of the LSTM, which controls how much of the internal cell state is exposed to the hidden state at time step *t*.

##### CNN–LSTM Model

The CNN–LSTM model takes input sequences of shape (F, 1). It begins with a Conv1D layer (64 filters, kernel size =5) followed by MaxPooling to extract local patterns. The output is then passed to an LSTM layer (64 units) to capture temporal dependencies. Finally, dense and dropout layers are applied before the softmax output layer. This hybrid approach employs convolutional layers for spatial feature extraction and LSTM layers for sequential modelling, making it suitable for tasks in which both local and temporal patterns are essential.

##### Classifier Head and Loss Function

For multi-class classification, the output of the LSTM or CNN–LSTM block is passed through a dense layer with softmax activation to produce class probabilities:

$$\hat{y}=\mathrm{softmax}\left(W_{\mathrm{clf}}h_{T}+b_{\mathrm{clf}}\right),$$

where $h_{T}$ is the final hidden representation, and $W_{\mathrm{clf}},b_{\mathrm{clf}}$ are the parameters of the classification layer. The softmax function is defined as:

$$\mathrm{softmax}\left(z_{i}\right)=\frac{e^{z_{i}}}{\sum_{j=1}^{n}e^{z_{j}}}.$$

The model is trained using the categorical cross-entropy loss:

$$L=-\sum_{i=1}^{n}y_{i}\log\left({\hat{y}}_{i}\right),$$

where $y_{i}$ is the true one-hot encoded label and ${\hat{y}}_{i}$ is the predicted probability for class *i*.

#### 2.4.8. Federated Learning Framework

To address data privacy and decentralisation constraints commonly encountered in healthcare environments, the CNN–LSTM model was trained using a federated learning strategy. After GA-based feature selection was completed centrally, the reduced feature representation was distributed to multiple clients (e.g., hospitals or healthcare centres), each holding local patient data.

Training proceeded over multiple communication rounds using the Federated Averaging (FedAvg) algorithm. At each round, the central server broadcast the current global model parameters to all clients. Each client performed local training on its private data and returned updated model parameters to the server. The server then aggregated these updates as:

$$w_{t+1}=\sum_{k=1}^{K}\frac{n_{k}}{N}w_{t}^{\left(k\right)},$$

where $w_{t}^{\left(k\right)}$ denotes the parameters learned by client *k*, $n_{k}$ is the number of local samples, and *N* is the total number of samples across all clients.

This process enables collaborative model learning without sharing raw patient data, preserving privacy while maintaining competitive diagnostic performance.

Federated clients were simulated by partitioning the dataset into multiple subsets, each representing a healthcare institution. Both IID and non-IID splits were explored, with non-IID partitioning introducing varying class proportions across clients to approximate inter-hospital heterogeneity.

## 3. Results

This section describes the experimental setup, baseline methods, training protocol, and evaluation metrics used to assess the performance of the proposed GA–LSTM framework for typhoid fever detection.

### 3.1. Baseline Methods

To rigorously assess the benefit of the proposed approach, several baseline models were implemented:

- **Logistic Regression**: A linear classifier using all features without feature selection.
- **Support Vector Machine (SVM)** with radial basis function (RBF) kernel.
- **Random Forest**: An ensemble of decision trees optimised via grid search.
- **XGBoost**: Gradient-boosted decision trees, representing a strong traditional ML baseline.
- **Plain BiLSTM**: BiLSTM trained on the full feature set without GA-based feature selection.

These baselines were chosen because they represent standard approaches in clinical ML and have been widely adopted in related typhoid and infectious disease prediction studies.

### 3.2. Training Protocol

For all models, training was performed on the training set, and tuning was performed on the validation set. Hyperparameters were selected using grid or random search strategies. For the LSTM, the number of layers, hidden units, dropout rate, learning rate, and batch size were tuned to balance performance and overfitting. Early stopping was applied based on the validation loss or the F1 Score.

For the GA-based feature selection, the population size, number of generations, crossover rate, and mutation probability were set based on preliminary experiments to achieve a stable trade-off between computational cost and search quality. Similarly, the GA for ensemble weight optimisation was run for a limited number of generations with a small population due to the low-dimensional search space.

### 3.3. Evaluation Metrics

Performance was evaluated using standard classification metrics suitable for imbalanced medical datasets:

- **Accuracy (ACC)**: Proportion of correctly classified samples.
  $$\mathrm{ACC}=\frac{TP+TN}{TP+TN+FP+FN}$$
- **Precision (PREC)**: Proportion of predicted positive cases that are truly positive.
  $$\mathrm{PREC}=\frac{TP}{TP+FP}$$
- **Recall (Sensitivity, SEN)**: Proportion of true positive cases correctly identified.
  $$\mathrm{SEN}=\frac{TP}{TP+FN}$$
- **Specificity (SPE)**: Proportion of true negative cases correctly identified.
  $$\mathrm{SPE}=\frac{TN}{TN+FP}$$
- **F1-score**: Harmonic mean of precision and recall.
  $$F1=2\times \frac{\mathrm{PREC}\times \mathrm{SEN}}{\mathrm{PREC}+\mathrm{SEN}}$$
- **Area Under the ROC Curve (AUC)**: Measures the trade-off between true positive and false positive rates.

$$\mathrm{AUC}=\int_{0}^{1}\mathrm{TPR}\left(\mathrm{FPR}\right)\;d\left(\mathrm{FPR}\right)$$

where

$$\mathrm{TPR}=\frac{TP}{TP+FN},\;\mathrm{FPR}=\frac{FP}{FP+TN}.$$

Given the class imbalance, evaluation relied not only on accuracy but also on macro-averaged F1 score and recall, which weight all classes equally. Additional metrics including Matthews correlation coefficient and Cohen’s kappa were reported to further account for imbalance and chance agreement.

### 3.4. Implementation Details

All experiments were implemented in Python 3.12.13. Training was conducted on a workstation equipped with a modern GPU and 32 GB of RAM. CNN–LSTM training completed within several minutes per experiment, while inference required only milliseconds per patient, supporting real-time clinical use.

### 3.5. Evaluation of Machine Learning and Deep Learning Models Using All Features

This section aims to examine the efficacy of various machine learning (ML) and deep learning (DL) models when trained on the complete set of clinical data, without using any feature selection. The aim of this experiment was twofold: (i) to establish a reliable baseline for the identification of typhoid fever, and (ii) to evaluate potential enhancements in performance and efficiency achievable in the future through deep learning-based feature selection.

All models were trained using the entire feature space of the clinical dataset. To ensure equitable evaluation of the models, the preprocessing pipeline was applied to each model individually. We employed standard evaluation metrics, including accuracy, macro-averaged precision, recall, F1-score, and macro-AUC (one-vs.-rest), to analyse the model’s performance on a held-out test set. This study analysed traditional machine learning classifiers and sophisticated deep learning models.

Two linear models, Logistic Regression and linear support vector machines, performed well. This shows that it is not difficult to distinguish between typhoid and non-typhoid cases in the original feature space. However, these models are more computationally expensive and require more memory because they must process all dimensions of the data.

Deep learning architectures enhance modelling capability by capturing nonlinear relationships among clinical features. This facilitates accurate predictions. The CNN–LSTM, presented in Table 2, and BiLSTM models achieved the highest test accuracies among the models tested. The CNN–LSTM model got a score of 91.02%. LSTM, BiLSTM, GRU, and BiGRU are all examples of recurrent architectures that performed well in general. This indicates that they can be applied to structured medical data.

Nevertheless, when all of the factors are taken into account, the difference in performance between ensemble machine learning models and deep learning models is still not that big. The results of this study indicate that deep learning models, despite their powerful capacities, are not as effective as they could be because they include features that are either unnecessary or only minimally related to the entire dataset.

Table 3 summarises the performance of representative ML and DL models trained on the complete feature set.

The results show that both machine learning and deep learning models can achieve strong baseline performance by using all relevant features. This leads to accuracies that are near to or even higher than 90%. Nevertheless, the difference in performance across different model families is really small. This suggests that the full feature space contains information that is not useful for decision-making.

CNN–LSTM is the most accurate model; however, the gap between it and ensemble machine learning approaches is not very big. Building deep learning models that use all features also requires more processing power, memory, and time for training and inference.

These results suggest that feature selection methods should be used to reduce the dimensionality of the data while maintaining or improving classification accuracy. We show in the next section that using GA-based feature selection not only preserves accuracy but also makes the process faster and easier to interpret. This is done by finding the fewest clinically important traits.

Although multiple machine learning and deep learning models were evaluated using the full feature set to establish a baseline, the CNN–LSTM architecture was selected as the reference classifier for subsequent feature selection. This is a really crucial point to make. Although performance differences across model families are relatively small, the CNN–LSTM consistently achieved the highest accuracy and stability, motivating its selection as the reference classifier. It could also find both local feature patterns using convolutional layers and complex dependencies using recurrent modelling.

As a result, CNN–LSTM was used as the primary classifier during feature selection using the genetic algorithm. This was done to test different sets of candidate features and then use the results to improve the evolutionary process. Using the best-performing deep learning model as the fitness evaluator could immediately improve the feature selection process for the target deployment model. This ensured that the selected clinical features improved CNN–LSTM’s diagnostic performance while reducing unnecessary information and costs.

### 3.6. GA Convergence and Individual Performance

This section aims to examine the experimental outcomes derived from employing a CNN–LSTM classifier alongside genetic algorithm (GA)-based feature selection for the identification of typhoid fever. The primary aspects of this issue are (i) the behaviour of the genetic algorithm during convergence, (ii) the comparative performance of several genetic algorithm individuals, and (iii) the statistical analysis of the most often selected clinical criteria.

The genetic algorithm optimisation technique was implemented within a population of 20 individuals over 10 generations, as presented in Table 4. A CNN–LSTM classifier was employed to evaluate each individual for a potential array of clinical characteristics. The results indicate that the solutions promptly coalesced to provide high-performing outcomes. In the initial generation, few individuals achieved accuracy rate over 91%.

The ability of individuals across various age groups to perform at comparable levels demonstrates the robustness of the technique, indicating it is not reliant on a certain feature combination. Table 5 presents a compilation of notable persons identified throughout the evolution of the GA. This table enumerates the characteristics and assessment criteria selected by these individuals.

The best individual (Ind–D) achieved the highest fitness score by maximising classification accuracy while minimising the number of selected features. This confirms the effectiveness of the GA fitness formulation in balancing predictive performance and model compactness.

**Figure 3:**
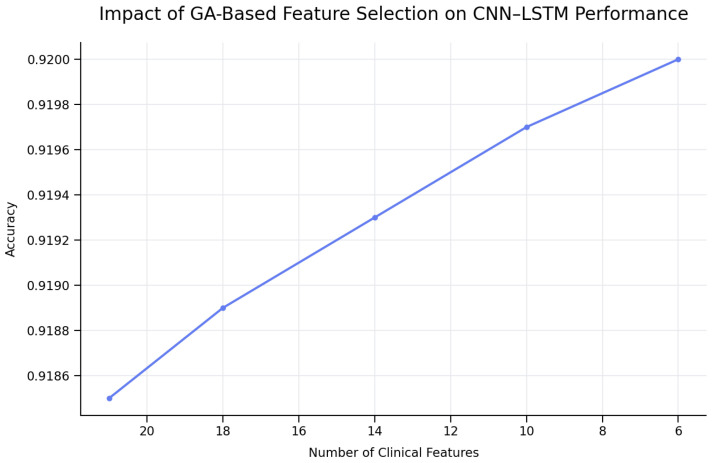
Impact of GA-based feature selection on CNN–LSTM diagnostic accuracy. The model maintains comparable performance while reducing the number of clinical features from 21 to 6, demonstrating effective dimensionality reduction without loss of accuracy.

As illustrated in Figure 3, GA-based feature selection enables a substantial reduction in the number of clinical parameters (21 → 6) while preserving diagnostic accuracy. This reduction significantly decreases computational complexity and memory requirements, which is particularly critical for medical decision-support systems and federated learning environments.

#### 3.6.1. Feature Selection Stability and Frequency Analysis

A detailed inspection of all GA individuals revealed that certain features were consistently selected across high-performing solutions. These features were among the most informative clinical parameters for typhoid detection.

The most frequently selected features included: *white blood cell count*, *skin manifestations*, *neurological symptoms*, *socioeconomic status*, and *blood culture result*. These parameters were present in nearly all individuals with accuracy above 91%, indicating their strong discriminative power.

Moderately frequent features such as *fever duration*, *platelet count*, *weather condition*, and *ongoing infection in society* contributed to performance improvements but exhibited redundancy with other clinical variables. In contrast, demographic features such as *age* and *gender* were selected less consistently, suggesting a lower predictive value in isolation.

The GA identified the following compact feature subset as optimal:

Gender, socioeconomic status, neurological symptoms, skin manifestations, white blood cell count, blood culture result.

Despite containing only six features, this subset achieved the same test accuracy (92.00%) as larger subsets with up to 14 features. This highlights the strong redundancy present in the original feature space and confirms the effectiveness of evolutionary feature reduction.

Table 6 presents the full evaluation metrics for the best-performing individuals. The best model maintains high Matthews Correlation Coefficient (MCC) and Cohen’s Kappa values, indicating strong agreement beyond chance and robust classification performance under class imbalance.

To assess robustness, the GA was executed multiple times with different random seeds. Across runs, the algorithm repeatedly converged toward compact feature subsets with comparable performance. Core features such as blood culture results, white blood cell count, and neurological symptoms were consistently selected, indicating stability of the feature selection process.

#### 3.6.2. Feature Selection Analysis

By removing unnecessary clinical features while maintaining diagnostic accuracy, the results showed that GA-based feature selection significantly improved the performance of CNN–LSTM. It was shown that identifying typhoid fever depended on a small set of clinically essential attributes rather than a large number of features, because the results were the same across many individuals.

The GA’s ability to reduce the number of features by more than 70% without sacrificing accuracy is important, as it makes the model easier to interpret, accelerates execution, and improves real-world applicability. The choice of laboratory and symptom-based criteria is congruent with what was previously known about how typhoid fever works in the body.

The GA–CNN–LSTM architecture described is a strong and simple way to use structured clinical data to find typhoid fever since it is easy to understand.

Medical decision-support systems must be able to make accurate predictions and operate efficiently. The CNN–LSTM model demonstrated strong classification performance even before being trained on all 21 clinical variables. On the other hand, the GA-based feature selection increased performance by a small but consistent amount, particularly in fitness score and consistency across runs. The test’s accuracy remained at 92.00%. Although the numerical improvement in accuracy is not particularly large, the reduction in the number of characteristics has a significant impact in practice.

Because the GA reduced the input feature space from 21 to six clinically significant parameters, the feature dimensionality was reduced by more than 70%. This enabled the use of less memory, the development of simpler models, and shorter training and prediction times. For deep learning architectures such as CNN–LSTM, fewer input features mean that the convolutional and recurrent layers have fewer parameters. This makes the process of reaching the end faster and less costly.

Diagnostic systems are often used in places with few resources, such rural clinics, embedded medical devices, or hospital decision-support platforms that work in real time. These improvements in efficiency are particularly important in the medical field, as diagnostic systems are often used in such settings. In many cases, improving accuracy is as critical as reducing memory usage and lowering processing latency. The GA-based feature selection proposed strikes a good balance: it preserves the model’s high diagnostic accuracy while significantly improving computational efficiency, making it better suited for real-world clinical settings.

Our study’s results show that evolutionary feature selection is not only a means of improving performance but also an important component of developing AI-based typhoid detection systems that are effective across a wide range of situations and can inform treatment.

#### 3.6.3. Feature Importance Analysis Using XGBoost

To validate the GA-based feature selection, an independent XGBoost model was trained on the original clinical features. Categorical variables were one-hot encoded, resulting in an expanded feature space of 55 variables corresponding to 21 clinical attributes. Aggregated feature importance was computed at the clinical variable level.

The analysis presented in Table 7 shows that blood culture results dominate prediction performance, accounting for approximately 89% of total importance. Other influential variables include complications, white blood cell count, neurological symptoms, and socioeconomic factors. Several of these variables, notably blood culture results and white blood cell count, overlap directly with the GA-selected feature subset.

XGBoost performs implicit feature selection by prioritising variables that produce the largest reduction in model loss during tree construction. As expected, laboratory confirmation via blood culture dominates feature importance due to its strong diagnostic specificity. However, reliance on a single dominant laboratory test may limit practical deployment in resource-constrained settings.

In contrast, the GA identifies a compact subset of six features that balances laboratory, clinical, and demographic information. While blood culture and white blood cell count are common to both methods, the GA additionally retains socioeconomic status, gender, and skin manifestations, which provide complementary diagnostic context. The strong overlap between GA-selected features and high-importance XGBoost variables supports the robustness of the GA approach, while its compactness enhances practical applicability for early screening and decentralised healthcare environments.

### 3.7. Comparison Between Centralised and Federated Learning Strategies

Table 8 presents a comprehensive comparison between centralised training and multiple federated learning strategies using the six GA-selected clinical features and a CNN–LSTM classifier. The centralised model achieves an accuracy of 92.00%, serving as an upper-bound reference where all patient data are directly accessible.

The accuracy, F1-score, MCC, and Cohen’s kappa of the centralised model are the same as those of the federated FedAvg model with IID client partitioning. This finding shows that federated learning can maintain diagnostic performance while keeping data private, provided that the clients’ data distributions are identical.

There is a little drop in performance when switching to a more realistic non-IID option. FedAvg achieves 91.94% accuracy with non-IID partitioning. This shows how differences in statistics between clients can change the results. This behaviour is to be expected in medical settings, where hospitals treat patients with diverse conditions. Even so, the performance loss is only slight, indicating that the proposed CNN–LSTM architecture is robust.

FedAvgM provides server-side momentum to accelerate convergence. In IID settings, the final accuracy remains the same as that of FedAvg. However, FedAvgM achieves the best validation performance earlier (round 10 rather than round 6), indicating that it helps stabilise and accelerate training.

FedProx was designed to prevent client drift in settings where IID is not used. Compared with FedAvg’s accuracy, FedProx’s ultimate accuracy of 91.75% is at the lower end of the scale. It does, however, converge more stably, as evidenced by a more even path of certification accuracy. When it comes to clinical deployments that are very different from one another, this trade-off is particularly important.

These results show that federated learning could work as well as centralised learning for diagnosing typhoid fever while keeping people’s privacy safe. Using federated CNN–LSTM modelling with GA-based feature selection yields a robust diagnostic framework that protects individuals’ privacy and can be applied in clinical settings.

**Figure 4:**
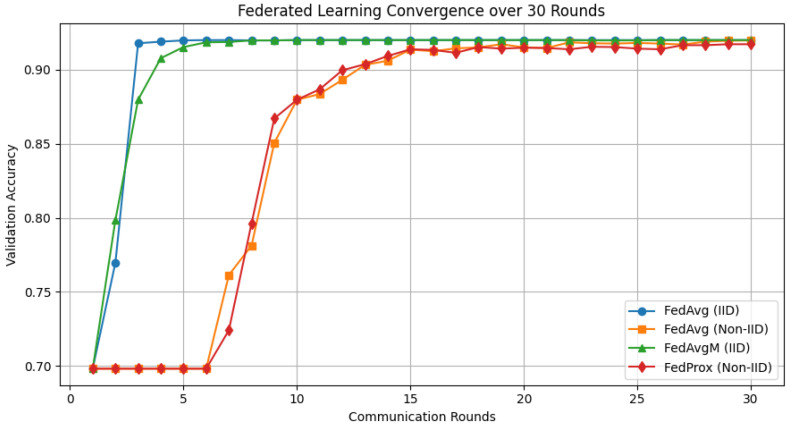
Validation accuracy convergence of centralised and federated CNN–LSTM models over 30 communication rounds using six GA-selected clinical features. IID-based methods (FedAvg and FedAvgM) converge rapidly within the first few rounds, while non-IID settings exhibit slower but stable convergence due to client data heterogeneity.

Figure 4 illustrates the convergence behaviour of different federated learning strategies over 30 communication rounds. In IID settings, both FedAvg and FedAvgM rapidly reach near-optimal validation accuracy within the first five rounds, demonstrating efficient aggregation when client data distributions are homogeneous. The inclusion of server-side momentum in FedAvgM further stabilises early updates, resulting in smoother convergence.

**Figure 5:**
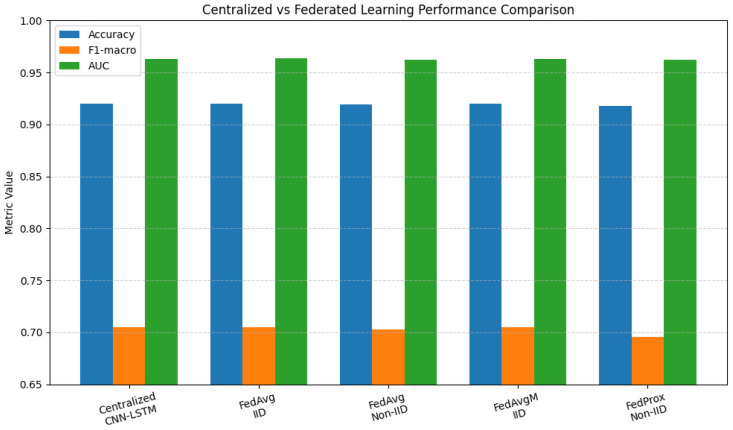
Final performance comparison between centralised and federated CNN–LSTM models using six GA-selected clinical features. The figure reports accuracy, macro F1-score, and AUC for centralised training and four federated learning strategies under IID and non-IID client distributions.

Figure 5 presents a comparative analysis of centralised and federated learning strategies using the CNN–LSTM model trained on the six GA-selected clinical features. The centralised model achieves an accuracy of 92.00%, representing the upper-bound performance when all data are centrally available.

Federated learning under IID client partitioning (FedAvg and FedAvgM) achieves identical accuracy and nearly identical F1-macro and AUC values, demonstrating that federated training can preserve diagnostic performance while enforcing data privacy. The negligible difference between centralised and federated IID results indicates that the proposed framework incurs almost no privacy-induced performance penalty.

In contrast, non-IID settings introduce a slight performance degradation. FedAvg under non-IID partitioning achieves an accuracy of 91.94%, whereas FedProx further reduces it to 91.75%. This behaviour reflects the impact of statistical heterogeneity across clients, which is representative of real-world clinical deployments where hospitals differ in patient populations and disease prevalence. Nevertheless, the reduction remains marginal, confirming the robustness of the proposed model.

Among federated strategies, FedAvgM demonstrates the best balance between convergence speed and final performance, achieving results identical to centralised training while benefiting from stabilised updates. Overall, these findings confirm that federated learning, when combined with GA-based feature selection and CNN–LSTM modelling, provides an effective privacy-preserving alternative to centralised medical data analysis without sacrificing diagnostic accuracy.

Importantly, all federated methods converge to a performance level comparable to centralised training, with final validation accuracy approaching 92%. This demonstrates that federated learning can effectively preserve diagnostic accuracy while enabling privacy-preserving collaboration across distributed medical institutions.

Independently of the federated client construction, the dataset exhibits significant class imbalance, with Normal or No Typhoid cases predominating, as shown in Table 9. As a result, accuracy alone may overestimate model performance. The use of macro-averaged metrics therefore provides a more reliable assessment of classification behaviour across all classes. The competitive macro-F1 scores indicate that the proposed framework maintains balanced diagnostic performance even under severe class skew.

### 3.8. Limitations

A limitation of this study is that federated clients were simulated from a single dataset rather than originating from independent hospitals. Although non-IID partitioning introduces a degree of statistical heterogeneity across clients, this setup does not fully capture the natural divergence observed in real multi-institutional environments. Consequently, validation on naturally diverged, multi-institutional datasets is required to fully assess federated learning behaviour under real-world deployment conditions.

With respect to data privacy, although federated learning enables collaborative model training without direct sharing of raw patient data, the standard federated averaging (FedAvg) strategy adopted in this study does not provide formal protection against inference attacks such as gradient leakage or model inversion. Accordingly, federated learning is regarded here as a privacy-preserving baseline rather than a complete privacy solution. Stronger privacy guarantees would require additional mechanisms, such as differential privacy or secure aggregation, which are beyond the scope of the present study and are identified as important directions for future work.

## 4. Conclusions

This study introduced a privacy-aware hybrid diagnostic framework for typhoid fever combining GA-based feature selection, CNN–LSTM modelling, and federated training. A key contribution is the identification of a compact six-feature subset that achieved competitive performance while reducing reliance on extensive laboratory testing. Unlike previous typhoid detection approaches based on centralised XGBoost or stacked metamodels using full feature spaces, the proposed method leverages deep sequential learning and decentralised training, making it well suited for deployment in resource-constrained and multi-institutional clinical settings.

We employed a CNN–LSTM architecture that utilised convolutional layers to capture local feature interactions and intricate correlations across clinical variables through recurrent modelling. The selected aspects were then incorporated into the design. The CNN–LSTM model, trained on a reduced feature set, performed comparably to models trained on the complete feature set, while incurring lower operational expenses, utilising less memory, and requiring less training time. This reduction is particularly significant in medicine, where the capacity to assimilate information rapidly and make prompt decisions is crucial.

The proposed architecture was evaluated in a federated learning context, in which multiple clients were collaboratively trained a CNN–LSTM model without disclosing raw patient data. The federated studies demonstrated that the model maintained performance comparable to that of centralised settings, regardless of IID or non-IID client distributions. This indicates that the strategy is robust and applicable in real-world healthcare environments, which are characterised by significant challenges related to data privacy and institutional data silos.

In the future, the framework will be enhanced to incorporate multi-class classification of febrile diseases. It will employ explainable artificial intelligence techniques to facilitate clinical interpretation and will undertake comprehensive validation studies across various centres to evaluate generalisability and clinical significance.

## Figures and Tables

**Table 1:** Summary of the clinical dataset used for evaluating the proposed framework.

Dataset	Samples	Features	Label Type	Number of Classes
Typhoid Multi-Class	31,087	21	Normal/Relapsing	4
			Acute/Complicated	

**Table 2:** CNN–LSTM training hyperparameters.

Model	Hyperparameter	Value
CNN–LSTM	Epochs	30
CNN–LSTM	Batch size	32
CNN–LSTM	Optimiser	Adam
CNN–LSTM	Learning rate	0.001

**Table 3:** Performance comparison of ML and DL models using all features.

Model	Accuracy	Precision (Macro)	Recall (Macro)	F1-Score (Macro)	AUC
Logistic Regression	0.9027	0.7578	0.7488	0.7483	0.9637
Random Forest	0.9095	0.7514	0.7481	0.7336	0.9638
Extra Trees	0.9078	0.7466	0.7471	0.7330	0.9634
XGBoost	0.9039	0.7593	0.7529	0.7504	0.9644
MLP (Sklearn)	0.8941	0.7572	0.7546	0.7557	0.9628
DNN	0.8975	0.7541	0.7511	0.7508	0.9627
LSTM	0.8982	0.7523	0.7483	0.7478	0.9612
BiLSTM	0.8971	0.7504	0.7468	0.7464	0.9620
CNN–LSTM	0.9102	0.7512	0.7454	0.7299	0.9623

**Table 4:** Genetic algorithm parameters.

Parameter	Value
Population size	20
Number of generations	10
Selection strategy	Tournament (k=3)
Crossover probability	0.9
Mutation probability	0.03
Minimum number of features	2
Fitness function	Validation accuracy with size penalty

**Table 5:** Performance comparison of representative GA individuals using CNN–LSTM. The best individual is highlighted in bold.

Individual	Gen.	Feat.	Accuracy	F1 (Macro)	AUC	Precision
Baseline	–	21	0.9102	0.7299	0.9623	0.7512
Ind–A	1	14	0.9127	0.6897	0.9621	0.8554
Ind–B	5	14	0.9200	0.7049	0.9630	0.8645
Ind–C	10	10	0.9200	0.7049	0.9628	0.8645
**Ind–D (Best)**	**Final**	**6**	**0.9200**	**0.7049**	**0.9633**	**0.8645**

**Table 6:** Comprehensive evaluation metrics for selected GA individuals. Best results are highlighted.

Individual	ACC	F1*_macro_*	MCC	Kappa	AUC	Prec*_w_*	Rec*_w_*	Feat.
Ind–A	0.9127	0.6897	0.8211	0.8012	0.9621	0.8554	0.9127	14
Ind–B	0.9200	0.7049	0.8395	0.8275	0.9630	0.8645	0.9200	14
**Ind–D**	**0.9200**	**0.7049**	**0.8395**	**0.8275**	**0.9633**	**0.8645**	**0.9200**	**6**

**Table 7:** Comparison of GA-selected features and aggregated XGBoost feature importance.

Clinical Feature	Selected by GA	XGBoost Importance	Interpretation
Blood Culture Result	✓	0.894	Confirmatory diagnostic test
White Blood Cell Count	✓	0.016	Bacterial infection marker
Neurological Symptoms	✓	0.0036	Clinical manifestation
Socioeconomic Status	✓	0.0031	Epidemiological risk indicator
Skin Manifestations	✓	0.0010	Supportive symptom
Gender	✓	0.0010	Demographic modifier
Complications	✕	0.044	Disease severity indicator

*Note:* ✓ indicates a feature selected by the genetic algorithm (GA), while ✕ indicates a feature not selected by GA.

**Table 8:** Comparative evaluation of centralised and federated learning strategies using six GA-selected features and a CNN–LSTM classifier.

Method	Client Split	ACC	F1*_macro_*	AUC	MCC	Kappa	Best Round	Privacy
Centralized CNN–LSTM	–	0.9200	0.7049	0.9629	0.8395	0.8275	–	No
FedAvg	IID	0.9200	0.7049	0.9638	0.8395	0.8275	6	Yes
FedAvg	Non-IID	0.9194	0.7026	0.9623	0.8381	0.8260	30	Yes
FedAvgM	IID	0.9200	0.7049	0.9631	0.8395	0.8275	10	Yes
FedProx	Non-IID	0.9175	0.6955	0.9624	0.8341	0.8218	29	Yes

**Table 9:** Dataset class distribution.

Typhoid Category	Description	Relative Frequency
Normal or No Typhoid	Negative or non-typhoid febrile illness	High (≈70–75%)
Acute Typhoid Fever	First episode of confirmed typhoid	Moderate (≈15%)
Relapsing Typhoid	Recurrent infection	Low (≈8%)
Complicated Typhoid	Severe outcome (e.g., sepsis, meningitis)	Very low (≈3–5%)

## Data Availability

The data used in this study are openly accessible at the following link: https://www.kaggle.com/datasets/rajmohnani12/typhoid-dataset (accessed on 10 January 2026).
